# The Prevalence of Endoparasites of Free Ranging Cats (*Felis catus*) from Urban Habitats in Southern Poland

**DOI:** 10.3390/ani10040748

**Published:** 2020-04-24

**Authors:** Izabela A. Wierzbowska, Sławomir Kornaś, Aleksandra M. Piontek, Kaja Rola

**Affiliations:** 1 Institute of Environmental Sciences, Faculty of Biology of Jagiellonian University, 7 Gronostajowa Str., 30-387 Kraków, Poland; i.wierzbowska@uj.edu.pl; 2 Faculty of Animal Breeding and Biology, University of Agriculture in Kraków, 24/28 Mickiewicza Av., 30-059 Kraków, Poland; s.kornas@ur.krakow.pl; 3 Institute of Botany, Faculty of Biology of Jagiellonian University, 3 Gronostajowa str., 30-387 Kraków, Poland; kaja.skubala@uj.edu.pl

**Keywords:** domestic cat, *Felis catus*, disease transmission, *Toxocara cati*, endoparasites, urban ecosystems, PCA, prevalence

## Abstract

**Simple Summary:**

Free ranging domestic cats are common in urban and suburban habitats and may cause a threat of disease transmission to other pets, wildlife and humans. We investigated the occurrence of endoparasites in cats in Kraków city, Southern Poland, based on examination of road-killed individuals. More than half of the cats were infected with at least one of seven identified parasites. Cats from suburban areas were more likely to be infected than cats from the city core. Our study supports the results from other studies, showing that cats may serve as a significant source of zoonotic transmission.

**Abstract:**

Growing urbanization leads to an increased risk of parasite spread in densely inhabited areas. Free-ranging cats can be locally numerous and come into frequent contact with both wildlife and humans. Cats are thus expected to contribute to parasitic disease transmission. In our study, we investigated the prevalence of endoparasites in free ranging cats in urban areas of Kraków city, based on necropsy of road-killed cats in relation to sex and diet of cat, season and habitat type. We found that 62% of 81 cats were infected with endoparasites with *Toxocara cati* being the most prevalent. In total, we identified seven parasite species. The number of parasite species was higher in suburban habitats and aside from *Eucoleus aerophilus* the prevalence of all parasites was higher in cats from suburban areas than in the individuals living in the city urban core. The prey of examined cats included mostly rodents, followed by soricomorphs and birds, which can all serve as paratenic hosts. Based on our results, we suggest that cats in urban areas should be considered as a serious potential zoonotic threat. Implementation of proper veterinary control and wider education on the topic is recommended.

## 1. Introduction

Environmental parasitic contamination depends on several factors including global warming, increased industrialization, urbanization and biodiversity loss [1,2]. It is assumed that pathogens are likely to thrive in the near future, mainly due to increased urbanization and growth of the human population. The dominant pathogens will be within the group of which transmission is density-dependent and/or of hosts, which are well adapted to urban environments [2]. In addition, higher temperatures in urban areas and overall global warming might increase for some parasites their growth, reproduction and even resilience [3,4,5]. Currently over 4 billion people live in urban areas, which accounts for 55% of the population; by 2030, it is estimated that the urban human population will rise to 68% meaning that two-thirds of the world’s population will live in cities [6]. Consequently, millions of people are also expected to migrate, often accompanied by pets. Density-dependent transmission of zoonotic parasites is expected to increase with high population densities and ownership of companion animals. Increased travel activity of people and animals all over the world may bring the possibility of transmission of many parasites, including exotic ones [7,8,9].

Among pets, the domestic cat (*Felis catus*) is one of the most common and widely distributed in the world [10]. According to FEDIAF (The European Pet Food Industry) [11] it has been estimated that Europe is inhabited by over 103 million cats and the European Union countries by over 75 million. Over 6 million cats live in Poland alone with 32% of households owning at least one cat [11]. Cats play an important role as companion animals but also are traditionally used to control rodent pests, especially in rural areas and in many developing countries [12,13]. Many cats have outdoor access to roam freely. This can pose a potential multidimensional health threat. Cats can contaminate the environment with various pathogens including parasitic, bacterial, fungal and viral elements that can be transmitted to humans [1,2,14]. Moreover, interactions with sympatric wildlife may result in spillover of parasites from domestic cats [14]. For example, *Bartonella* spp., *Toxoplasma gondii* and feline immunodeficiency virus (FIV) can be transmitted from domestic cats to mountain lions (*Puma concolor*) and bobcats (*Lynx rufus*) [15] while feline leukemia virus (FeLV) is transmittable to Iberian lynx (*Lynx pardinus*) [16].

Cats can be infected by numerous endoparasites, including protozoa and helminths [17,18] that not only cause diseases in cats but also are of zoonotic significance. More than 75% of human disease is of zoonotic origin and from both wildlife and domestic animals [19]. For example, *T. gondii*, which is probably the most widespread and prevalent parasitic protozoan of major importance to public health. The prevalence of this zoonotic parasite is reported worldwide [13,14,15]. One of the possible infection routes is by ingestion of oocysts, which are shed by domestic and wild felids [20].

In the case of nematodes such as *Toxocara cati* (which are responsible for human visceral larva migrans VLM and ocular larva migrans OLM), ingestion by humans of eggs or larvae can lead to zoonotic toxocarosis [17,21]. Eggs of *T. cati* are dispersed via the animal’s feces in the environment and mature in soil [7]. Eggs of *Toxocara* spp. are the most frequently found helminth eggs in diagnostic fecal samples of dogs and cats in Germany [8]. The contamination by the eggs of geohelminths such as *Toxocara, Ascaris, Trichuris* and *Ancylostoma* can reach up to 40% of sandy playgrounds in Hanover [22] and recreational areas in Poland [23]. Additionally, in Poland, seroprevelance of human toxocarosis has been reported in up to 75.6% sera samples [24]. Humans may also become infested with zoonotic cestodes from cats such as *Dipylidium caninum* or *Echinococcus multilocularis* [1,18]. There are many reports of *D. caninum* infections in children due to ingestion of infected fleas [17].

Overall, the prevalence of endoparasites of cats in Europe has been found to vary between 20% and 40% [17]. Several studies report that stray or free-ranging outdoor cats have a higher frequency of parasites than indoor kept cats [8,14,17]. In Greece stray cats were 8.8 times more likely to be infected with Ancylostomatidae when compared to owned cats. Similarly, the infection of *T. cati* was 2.7 times higher for cats living outdoors in comparison to those staying indoors [7]. In addition, cats represent a more important potential reservoir of parasites than domestic dogs [25].

Domestic cats can be infected by parasites whose life cycles involve transmission from the soil, prey or other carnivores [1,14,18]; consequently they can serve as a potential threat to other companion animals, wildlife and people living in the same environment. Therefore, it is of great importance to carry out further research into parasitic infections in domestic cats. Urban and peri-urban areas should be of particular interest in the context of transmission of parasitic diseases. Both environments can be attractive to wild animals, which can easily adapt and participate in parasitic vector-borne infections. For example, the stone marten (*Martes foina*) or red fox (*Vulpes vulpes*) can play an important role for transmission of echinococcosis and capillarosis to companion animals [1,26,27].

The aim of this study was to investigate the prevalence of endoparasites in outdoor cats in urban areas of Kraków city. The present study provides analyses of potential factors such as age and sex of the cat as well type of the habitat and diet of the cats, which might reflect the infestation of specific parasites.

## 2. Materials and Methods

### 2.1. Study Area

The study was conducted on domestic cats collected as road kills in Kraków metropolitan area, in Southern Poland. Kraków is the second largest city in Poland with a total surface area of 327 km^2^ and human population over 769,000 (as stated for year 2018) [28]. It is also a popular tourist destination with 7.6 million visiting tourists per year. Built-up and urbanized areas constitute over 46% of the city. The city core is strongly urbanized while the peripheral districts are mostly composed of blocks of flats and detached houses with patches of green areas. Green areas are more dominant in suburban parts of the city and a large proportion of the city is still used for agricultural purposes with arable land, orchards, meadows and pastures covering 44% of total city area. Thus, we classified the locations of collected road killed cats into urban and suburban habitats. The estimated number of stray cats in Kraków is over 2400 [29].

### 2.2. Data Collection and Parasitological Analysis

Data were obtained from road-killed cats (*n* = 81), which had been collected by the “Wild Rescue” company (‘Dzikie Pogotowie Maciej Lesiak’) with the permission of the local municipality. Animals were collected between November 2011 and April 2013. Cats were weighed with precision of ± 1 g. We identified sex and age (based on teeth wear) of the individuals. A total of 81 cats (53 males and 28 females) were examined. There were 49 cats in age group 1–2 years old and 32 individuals aged above 2 years. All collected cats were examined by necropsy. Prior to examination all samples were frozen at −85 °C for at least two weeks [30] in order to stop development of parasitic forms dangerous to humans, namely *Echinococcus* sp., which occurs more frequently in Poland especially in the red fox [31]. Necropsies were made according to the method appropriate for parasitological study, e.g., [32,33]. We checked the abdominal cavity and the internal organs including stomach, digestive system, kidney, liver, heart and lungs (scraping of the mucosa of intestine, cutting the organs into small pieces and submerging them in pepsin enzyme). The fecal samples for presence of Protozoan oocysts were examined using the McMaster method with saturated sugar solution. The parasites were preserved in 75% ethanol with 5% glycerol addition, then were mounted and cleared with lactophenol. Tapeworms were identified to the lowest possible taxonomic level. Parasites biodiversity was determined based on morphological features [34]. The parasitic infection was expressed as prevalence of infection (number of cats infected with parasites/total number of examined cats × 100%) and intensity of infection (mean number of parasite individuals per an infected cat).

### 2.3. Diet Analysis

Out of 81 cats’ stomachs, 63 contained food remains, and the rest were empty or contained only cat hair. Dried stomach content was weighed and divided into the following food categories: rodents, soricomorphs, birds, invertebrates, anthropogenic food and plant material. Prey species were identified based on remains of hair, feathers and bones using keys and reference material stored as a collection belonging to the Institute of Environmental Sciences Jagiellonian University. Mammals were identified to species or genus based on their bones, teeth [35] and hair [36]. Birds were identified to order based on feathers [37]. Diet of cats was expressed as frequency of occurrence in stomachs %FO (number of stomachs with one food category/total number of examined stomachs × 100%).

### 2.4. Statistical Analysis

The 95% confidence intervals (CI) of the prevalence values were estimated according to [38] including continuity correction. The non-parametric Mann–Whitney U test was performed in order to reveal significant differences in the intensity of infection of the two most common parasites (i.e., *Toxocara cati* and *Taenia taeniaeformis*) between urban and suburban areas. Separate principal component analyses (PCA) based on correlation matrix were applied to show the association of parasites to particular groups of cats. The groups included division of cats according to: (1) sex and age, (2) sex and season, (3) sex and habitat and (4) habitat and age. Matrices for analyses consisted of averaged frequencies of a given parasite in particular groups. The Mantel test [39] was performed to determine the relationship and statistical correlation between the two matrices of similarity, i.e., cats’ diet similarity matrix and parasite similarity matrix. The input matrices were based on the mean frequencies of food categories/parasites in groups of cats for particular combinations: season × sex × age. Finally, a seriation of all parasites (presence/absence values) recorded in cats sorted according to their weight was performed using a constrained algorithm [40]. The statistical calculations were performed using PAST 3.25 [41].

## 3. Results

### 3.1. Prevalence of Endoparasites

Overall parasites were detected in 50 (=62%) out of the 81 cats. Five cats (6%) were infected by one parasite. No *Echinococcus multilocularis* or *Cystoisospora* were detected in the examined cats. *Toxocara cati* was the most prevalent parasite in the examined cats (*n* = 36, 44.44%), followed by *Taenia taeniaeformis* (*n* = 34, 41.98%), *Ancylostoma tubaeformae* (*n* = 17, 20.99%), *Dipylidium canininum* (*n* = 5, 6.17%) and *Toxascaris leonina* (*n* = 3, 3.70%). *Mesocestoides* sp. was identified in two cats, similarly *Eucoleus aerophilus* (Table 1).

Overall 14.81% (12/81) of the examined cats had been infected by one parasite. Among other individuals 45 cats (55.56%), 11 (13.58%) and 1 (1.23%) harbored two, three and four different parasites, respectively (Table 2).

**Figure 1:**
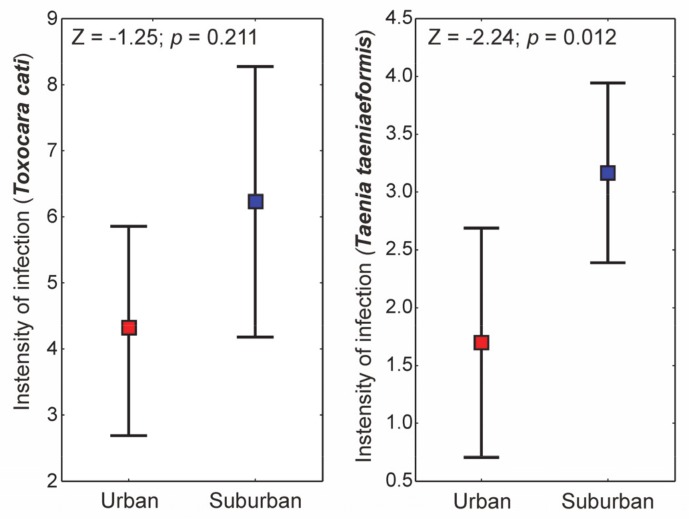
Whisker plots (mean ± SE) of intensity of infection in the two most common parasites of cats in urban and suburban areas. The results of Mann–Whitney U tests are provided in the inset of the graph.

The cats collected from urban and suburban areas of Kraków city were represented by 33 and 48 individuals respectively. As many as seven parasite species were detected in cats from the suburban area, whereas six were detected from the urban habitats. Apart from *Eucoleus aerophilus*, the prevalence of each parasite was greater in suburban compared to urban areas (Table S1). The intensity of infection with *Taenia taeniaeformis* proved to be significantly higher in cats collected from suburban area (Figure 1). The intensity of infection with the second most common parasite, i.e., *Toxocara cati,* was also higher in suburban areas compared to urban ones but no significant differences were found (Mann-Whitney test; *p* > 0.05).

### 3.2. Diet Analysis

In examined material, 63 cats had identifiable content in their stomachs, in which we identified 43 vertebrate prey items: rodents (three species), soricomorphs (two species) and birds (two orders), respectively (Table 3). The most frequently consumed prey species (31 individuals) was the common vole (*Microtus arvalis).* Other prey species were not numerous (no more than three individuals) and included the bank vole (*Myodes glareolus*), mice (*Apodemus* spp.), common shrew *(Sorex araneus),* pygmy shrew *(Sorex minutus)* and birds from Columbiformes and Passeriformes. Anthropogenic food was found in 65% of examined stomachs. The diet of the cats did not differ between males and females. Cats aged 1–2 years old consumed the majority of identified vertebrate prey items (37 of 43 prey individuals). Similarly cats collected in suburban areas consumed more prey than cats from the urban habitats (38 of 43 prey individuals; Table 3).

### 3.3. Differentiation in the Occurrence of Parasites in Cats and Its Relationship with Diet

**Figure 2:**
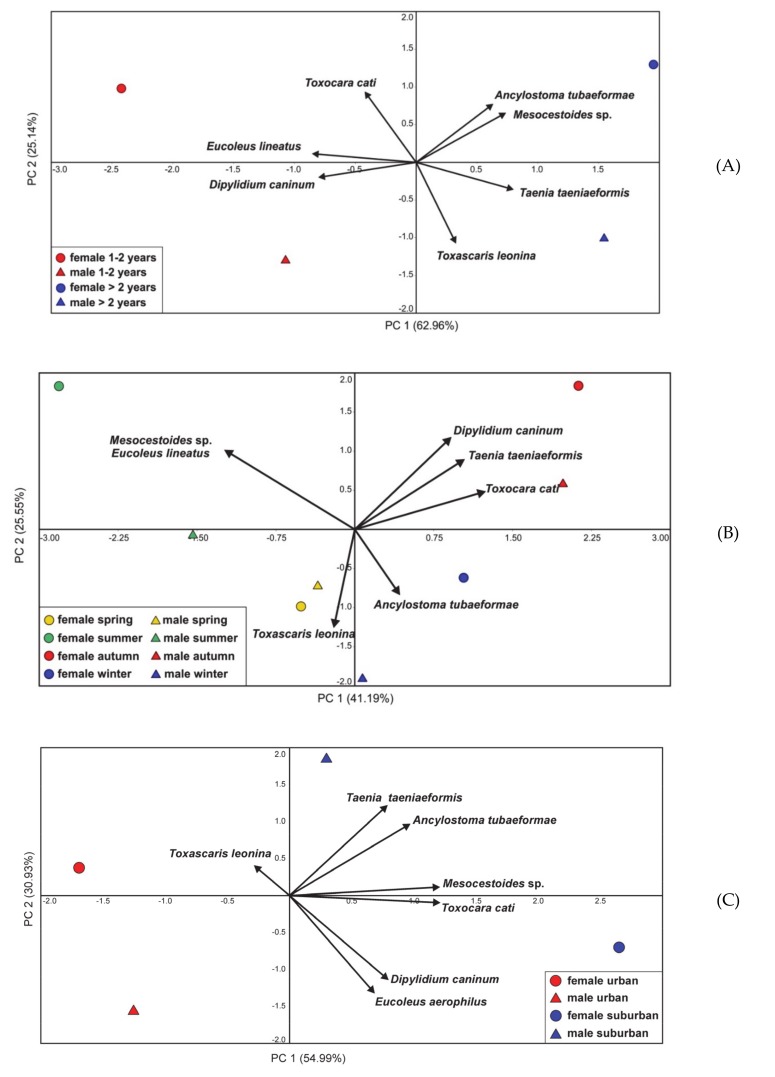

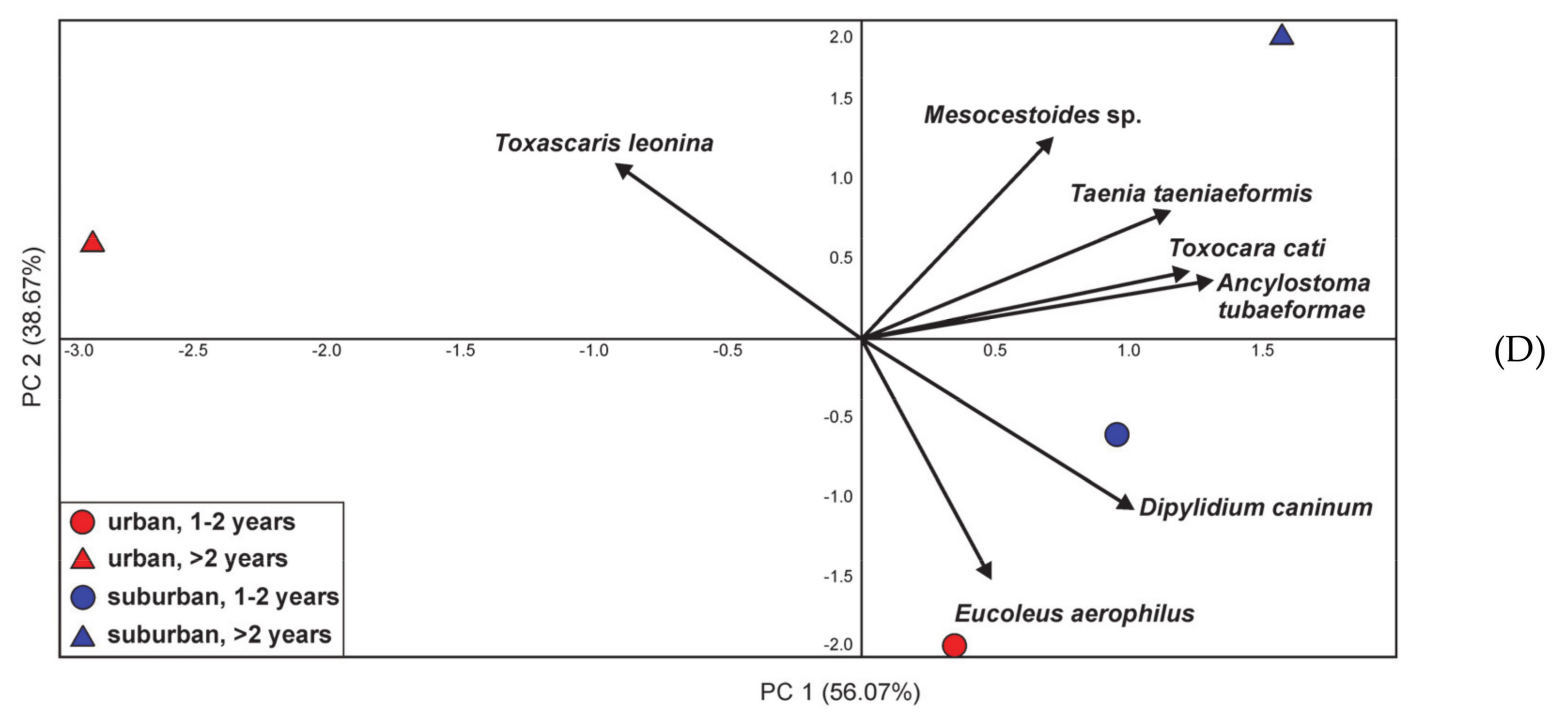
Biplot of the first two axes of the principal component analysis (PCA) of the groups of cats and parasites in relation to (**A**) cats’ sex and age, (**B**) cats sex and season, (**C**) cats sex and habitat and (**D**) habitat and cats age. The percentage of variance explained by the principal components is provided in parentheses.

Occurrence and frequency (%) values of endoparasite infections in cats in relation to age, sex, habitat and season are provided in the Supplementary Materials (Tables S1–S4). In general, *Eucoleus aerophilus* and *Dipylidium caninum* were more associated with young cats (1–2 years old) regardless of their sex, whereas *Taenia taeniaeformis* and *Mesocestoides* sp. occurred more frequently in both male and female adult cats (Figure 2A).

Regardless of the sex, parasite composition depended considerably on the season. *Mesocestoides* sp. and *Eucoleus aerophilus* were strongly associated with the summer season; and *Taenia taeniaeformis, Dipylidium caninum* and *Toxocara cati* with the autumn season (Figure 2B). With regards to sex of the examined cats and habitat where they were found, most of the parasites, with the exception of *Toxascaris leonina,* were more associated with male and female cats from a suburban habitat (Figure 2C). There was a clear association of *Eucoleus aerophilus* and *Dipylidium caninum* with younger cats regardless of the habitat they lived in, whereas *Mesocestoides* sp., *Taenia taeniaeformis*, *Ancylostoma tubaeformae* and *Toxocara cati* were mostly associated with older cats from suburban habitats (Figure 2D).

**Figure 3:**
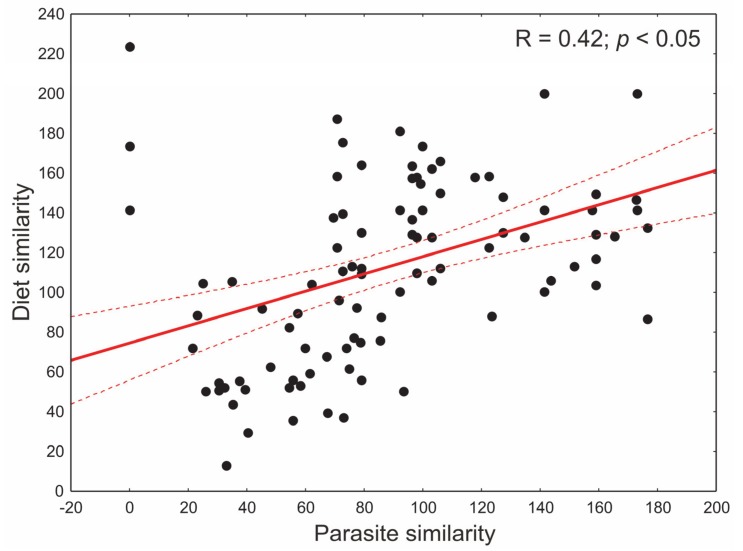
Results of the Mantel test. The correlation between cats’ diet similarity and parasite similarity. Pearson correlation coefficient as well as regression line with 95% confidence interval are provided.

The results of the Mantel test showed a significant correlation (R = 0.25, *p* < 0.05) between cats’ diet similarity and parasite similarity (Figure 3). This indicates that the similarity in parasite composition increased with increased similarity of cats’ diet.

**Figure 4:**
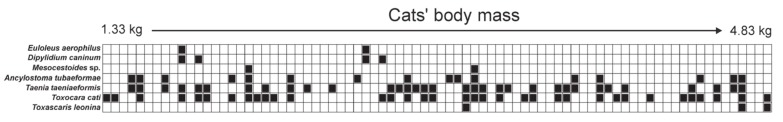
The seriation diagram using a constrained algorithm of parasites (presence/absence values) recorded in cats sorted according to their body mass.

We found no clear association between cats’ weight and presence of parasites (Figure 4).

## 4. Discussion

The present study provided a detailed view of the gastrointestinal parasitism of domestic cats in an urban area of Poland. Overall, the prevalence of parasitic infestation of the cats was 62%. Similarly, high levels of parasite prevalence were recorded in recent studies conducted worldwide. For example, the occurrence of infection in India (Mumbai) was 77.22% [42], in Kenya 73.2% [13], in United Arab Emirates (Dubai) 87% [43], in Iran 94% [44] and 57.9% in Malaysia [45]. In European countries the overall prevalence of parasite infestation of cats was 30.8% [46]. Lower prevalence levels were recorded in The Republic of Korea 39.8% [47], in the USA 24.5% [9] and Brazil 18.1% [48]. However, it is important to indicate the status of the cat. In general, stray or cats with outdoor access have higher infection levels in comparison to cats living indoor [14].

In the present study, *Cystoisospora* were not detected in examined cats. This could be explained by the age of the examined cats, which were at least 1 year old. Coccidiosis has higher prevalence in younger cats (6–12 months) [9,49] and in older cats lower prevalence is explained by acquired immunity and better resilience [50]. The most frequently recorded parasite in the examined cats was *Toxocara cati* with an overall prevalence 44.44%. It may suggest high distribution of *T. cati* eggs in outdoor environments. This ascarid is known for having a global distribution, which is mainly due to high resistance of eggs in extreme conditions [46]. Moreover, transmammary transmission increases infestation among young cats [51]. In our study both age groups of cats had a prevalence of this parasite over 40%. Information of such high prevalence of this zoonotic parasite is important with regard to public health. High prevalence of *T. cati* in cats might be explained by high predation and consumption of paratenic hosts such as rodents and birds [25]. In the examined cats the majority of prey were rodents including the common vole (*M. arvalis*), and *Apodemus* spp. followed by soricomorphs and birds. All may act as reservoirs for numerous parasites and consequently play an important role in circulation of infections among other hosts including cats and humans [1,2,43]. Species of rodents identified in our study (*M. arvalis, M. glareolus* and *Apodemus spp.*) have been reported to be infected by *Toxocara* spp. in urban habitats [52]. In peri-urban areas, the abundance of wildlife species is higher. Domestic cats, allowed to roam freely in such sites, are more vulnerable to being infected and participate in further transmission. In general, the parasitic prevalence in cats from suburban areas was higher than in the individuals living in the city urban core. In particular, infestation by *T. cati* was more associated with cats living in suburban areas indicating their outdoor access (Figure 2C,D).

The other ascarid infecting cats, *Toxascaris leonina*, was positive in 3.70% (*n* = 3 cats). The majority of studies from Europe have indicated a rather low prevalence of this nematode (0.1–0.3%) [7,17,53] but in Hungary, this nematode prevalence in examined cats was 7.2% [49]. In addition, *T. leonina* has been recorded in many canid and felid species. Cats can be infected by ingesting either eggs or rodents, which can play the role of optional intermediate host [54]. *T. leonina* has the potential to cause human disease as emerging zoonosis [1].

Among cestodes, a high prevalence (41.98%) was detected in cats of taeniid infection by *Taenia taeniaeformis*. In our study this parasite was associated more with older cats living in the suburbs. This tapeworm can use rodents in the life cycle, the dominant prey of cats in our study. This coincides with results that were shown by [43,49] who indicated that *T. taeniaeformis* had the lowest prevalence in the city centre compared to suburb districts and linked this fact to lower abundance of rodents.

It is also argued by [9,17,49] that detection of cestodes by coproscopy results in much lower prevalence range 1–3% (e.g., [9,46,53]) than necropsy: 60% [44] and 17% [43]. Thus, it is recommended to include this fact in further analysis, in particular as it is disputed that eggs from *Taenia* and *Echinococcus* cannot be distinguished and that “cats excreting taeniid eggs must be initially considered as possibly infected with *Echinococcus multilocularis*” [53].

The hookworm species detected in domestic cats was *Ancylostoma tubaeforme*. The overall prevalence of this parasite in cats was 20.99%. It occurred more frequently in suburban cats. This again could be associated with cats feeding on rodents, which serve as paratenic hosts of the parasite [18]. Hookworms are common parasites of cats and dogs but have a potential role as a source of zoonotic disease, being known as a cause of cutaneous larva migrans [55,56]. Our results are consistent with some studies of cats, e.g., the prevalence of ancylostomatidosis was 26% in Hungary [49] and 21% in the United Arab Emirates [43]. In India the prevalence of *Ancylostoma* spp. detected in cats was 52.78% and associated with mainly older individuals (above one year old) [42]. In the USA, Brazil, Kenya and several European countries the prevalence of hookworms ranged between 0.5% and 9.7% [7,9,13,21,46,48,53].

Tapeworms belonging to the species *Dipylidium caninum* (6.17%, *n* = 5 cats) were found at a lower prevalence compared to other parasites detected in the examined cats. This is consistent with other results [17,48,57]. However, there are reports of high prevalence of this parasite as in the studies conducted in Belgrade where 22% of cats were infected by *D. caninum* [21]. In the life cycle this cestode uses fleas as intermediate hosts. Humans can be infected by ingestion of infected fleas most commonly brought home by cats [18].

In the present study two parasite species *Mesocestodes* sp. and *Eucoleus aerophilus* were detected in low prevalence (both 2.47% *n* = 2 cats infected). *Mesocestoides* sp. are not commonly reported in literature and their prevalence in infected cats does not exceed 1% [9] but in Iran 78% of examined cats were positive for this parasite [44] The life cycle of *Mesocestoides* sp. is not clearly defined: it is possible cats get infected through ingestion of vertebrate intermediate hosts containing tetrathyridia [18].

In our study, we did not find pulmonary nematodes in the examined cats except for *Eucoleus aerophilus* (Trichurida, Trichinellidae, syn*. Capillaria aerophila*). Together with *D. caninum* these two parasites were more associated with younger cats (1–2 years old) regardless of the habitat they occupied. Consistent with research of [46] it is reported that wildlife species like the red fox, have a direct impact on dispersion of lung capillariosis in cats [27,49]. The infection of cats can be also explained by ingestion of paratenic hosts such as rodents, soricomorphs and birds [58]. In recent years, more attention has been paid to pulmonary nematodes due to their high pathogenicity [59]. Lung nematodes *Aelurostrongylus abstrusus* (Strongylida: Angiostrongylidae) were commonly reported in cats across Europe [27,46] and together with *E. aerophilus* are currently considered the most important causes of parasite-induced respiratory infection in felids [27]. Recently, the pulmonary nematode *Troglostrongylus brevior* (Strongylida: Crenosomatidae) has also been detected in domestic cats [60].

Although we analyzed the effect of season on the prevalence of parasites in cats, it must be emphasized that the examined cats could have been infected earlier and it is not possible to indicate the beginning of the parasitic infestation. In our study we recorded higher prevalence of parasitic infestation of examined cats in both summer and autumn seasons. This could be associated with better access to wildlife prey as well as the breeding season of the cats followed by a higher prevalence in newborn kittens [9]. Stray or outdoor cats are likely to be infested with parasites [7,9,14,25,43], however, even owned cats are at risk of being infested. A recent survey [8] revealed that domestic cats are commonly not dewormed properly by their owners who might be even reluctant to use anthelmintics as they are afraid of using chemical treatments. Moreover, pet owners often have insufficient knowledge about zoonoses transmitted by cats.

In the future, the expanding contact between humans, domestic animals and wildlife will likely have an impact on zoonotic helminth spread [61]. Considering that cats and dogs are the most numerous pets, and that often they are in very close contact with their owners, this should bring attention to the higher risk of possible transmission of zoonoses [21,61].

## 5. Conclusions

Our data indicate a high prevalence of endoparasite infections in free ranging cats in urban and suburban areas in Poland. *Toxocara cati* and *A. tubaeformae,* associated with zoonotic diseases, were among the most prevalent parasites in the examined cats. This should be considered important information about possible health risks and the need for implementation of proper veterinary control. Outdoor access and uncontrolled roaming of cats increases the risk of parasitic infections. This includes also cats that feed on small vertebrates. In suburban areas the probability of interspecific parasitic transmissions is more common in cats. Thus, it is crucial to educate the owners about possible exposure to zoonotic diseases through their pets.

## Figures and Tables

**Table 1:** Prevalence (%) and intensity of parasitic infections of studied cats.

Endoparasite	N Infected, Prevalence of Infection (%)	Mean Intensity of Infection	Range of Infection	95% C.I.	
				Lower	Upper
*Toxocara cati*	36 (44.44)	12.22	1–81	34.54	55.87
*Toxascaris leonina*	3 (3.70)	1.00	1	0.96	11.18
*Ancylostoma tubaeformae*	17 (20.99)	7.18	1–12	13.04	31.07
*Taenia taeniaeformis*	34 (41.98)	6.12	1–32	31.27	53.46
*Dipylidium caninum*	5 (6.17)	2.80	1–7	2.29	14.44
*Mesocestoides* sp.	2 (2.47)	1.00	1	0.43	9.46
*Eucoleus aerophilus*	2 (2.47)	2.50	1–4	0.43	9.46

**Table 2:** Prevalence (%) of mixed endoparasite infections in cats.

Mixed Endoparasite Infections	N Infected, Prevalence of Infection (%)	95% C.I.	
		Lower	Upper
*T. cati + T. taeniaeformis*	24 (29.63)	20.26	40.96
*T. cati + A. tubaeformae*	10 (12.35)	6.40	21.99
*T. cati + T. leonina*	1 (1.23)	0.06	7.63
*T. cati + D. caninum*	3 (3.70)	0.96	11.18
*T. cati + Mesocestoides* sp.	2 (2.47)	0.43	9.46
*T. cati + E. aerophilus*	1 (1.23)	0.06	7.63
*T. cati + T. taeniaeformis + A. tubaeformae*	10 (12.35)	6.40	21.99
*T. cati +T. leonina + T. taeniaeformis*	1 (1.23)	0.06	7.63
*T. cati + T. leonina + A. tubaeformae + T. taeniaeformis*	1 (1.23)	0.06	7.63
*T. taeniaeformis + A. tubaeformae*	4 (4.94)	1.59	12.84

**Table 3:** Food categories in cats’ diet based on examined stomachs. *n*—number of stomachs with content, FO—number of stomachs containing food category.

Food Categories	Urban *n* = 26		Suburban *n* = 37		Total *n* = 63	
	FO	%FO	FO	%FO	FO	%FO
rodents	4	15.4	11	29.7	15	23.8
soricomorphs	-	-	3	8.1	3	4.8
birds	2	7.7	3	8.1	5	7.9
invertebrates	2	7.7	3	8.1	5	7.9
anthropogenic food	20	76.9	21	56.8	41	65.1
plant material	12	46.2	23	62.2	35	55.6

## Supplementary Materials

The following are available online at https://www.mdpi.com/2076-2615/10/4/748/s1, Table S1: Occurrence, mean intensity, and prevalence (%) with 95% CI of parasitic infections of cats in different habitat types; Table S2: Occurrence, mean intensity, and prevalence (%) with 95% CI of parasitic infections of cats in different sex groups; Table S3: Occurrence, mean intensity, and prevalence (%) with 95% CI of parasitic infections of cats in different age groups; Table S4: Occurrence, mean intensity, and prevalence (%) with 95% CI of parasitic infections of cats in different seasons.
