# Supplementary material for: The Prevalence of Endoparasites of Free Ranging Cats (Felis catus) from Urban Habitats in Southern Poland
Source: Animals (Basel). 2020 Apr 24;10(4):748. doi: 10.3390/ani10040748 (PMC7222823; doi:10.3390/ani10040748)
Supplement: Supplementary file 1 [file animals-10-00748-s001.pdf]

Table S1. Occurrence, mean intensity, and prevalence (%) with 95% CI of parasitic infections of cats in different habitat types.

Endoparasite infections	Kraków urban (n=33)					Kraków suburban (n=48)				
	N infected	Prevalence of infection (%)	Mean intensity of infection	95% CI		N infected	Prevalence of infection (%)	Mean intensity of infection	95% CI	
				lower	upper				lower	upper
Toxocara cati	11	33.33	4.27	18.55	51.89	25	52.08	6.23	37.35	66.48
Toxascaris leonina	1	3.03	0.03	0.16	17.51	2	4.17	0.04	0.73	15.43
Ancylostoma tubaeformae	4	12.12	0.76	3.96	29.14	13	27.08	2.02	15.74	42.09
Taenia taeniaeformis	8	24.24	1.70	11.74	42.63	26	54.17	3.17	39.31	68.37
Dipylidium caninum	2	6.06	0.06	1.06	21.62	3	6.25	0.25	1.63	18.21
Mesocestoides sp.	0	0.00	0.00	0.00	12.98	2	4.17	0.04	0.73	15.43
Eucoleus aerophilus	1	3.03	0.03	0.16	17.51	1	2.08	0.08	0.11	12.46

Table S2. Occurrence, mean intensity, and prevalence (%) with 95% CI of parasitic infections of cats in different sex groups.

Endoparasite infections	Female (n=28)					Male (n=53)				
	N infected	Prevalence of infection (%)	Mean intensity of infection	95% CI		N infected	Prevalence of infection (%)	Mean intensity of infection	95% CI	
				lower	upper				lower	upper
Toxocara cati	14	50.00	8.21	31.07	68.93	22	41.51	3.96	28.42	55.82
Toxascaris leonina	0	0.00	0.00	0.00	15.02	3	5.66	0.06	1.47	16.63
Ancylostoma tubaeformae	6	21.43	1.43	9.03	41.46	11	20.75	1.55	11.29	34.49
Taenia taeniaeformis	11	39.29	3.82	22.13	59.27	23	43.40	1.91	30.11	57.65
Dipylidium caninum	2	7.14	0.39	1.25	24.95	3	5.66	0.06	1.47	16.63
Mesocestoides sp.	1	3.57	0.04	0.19	20.24	1	1.89	0.02	0.10	11.38
Eucoleus aerophilus	1	3.57	0.14	0.19	20.24	1	1.89	0.02	0.10	11.38

Table S3. Occurrence, mean intensity, and prevalence (%) with 95% CI of parasitic infections of cats in different age groups.

Endoparasite infections	Age 1-2 years (n=49)					Age > 2 years (n=32)				
	N infected	Prevalence of infection (%)	Mean intensity of infection	95% CI		N infected	Prevalence of infection (%)	Mean intensity of infection	95% CI	
				lower	upper				lower	upper
Toxocara cati	22	44.90	7.08	30.94	59.66	14	43.75	2.91	26.84	62.12
Toxascaris leonina	1	2.04	0.02	0.11	12.24	2	6.25	0.06	1.09	22.22
Ancylostoma tubaeformae	10	20.41	1.41	10.73	34.76	7	21.88	1.66	9.95	40.45
Taenia taeniaeformis	19	38.78	2.73	25.55	53.76	15	46.88	2.31	29.51	64.97
Dipylidium caninum	4	8.16	0.27	2.65	20.48	1	3.13	0.03	0.16	18.01
Mesocestoides sp.	0	0.00	0.00	0.00	9.06	2	6.25	0.06	1.09	22.22
Eucoleus aerophilus	2	4.08	0.10	0.71	15.14	0	0.00	0.00	0.00	13.34

Table S4. Occurrence, mean intensity, and prevalence (%) with 95% CI of parasitic infections of cats in different seasons.

Endoparasite infections	Spring (n=36)					Summer (n=31)					Autumn and Winter (n=14)				
	N infected	Prevalence of infection (%)	Mean intensity of infection	95% CI		N infected	Prevalence of infection (%)	Mean intensity of infection	95% CI		N infected	Prevalence of infection (%)	Mean intensity of infection	95% CI	
				lower	upper				lower	upper				lower	upper
Toxocara cati	14	38.89	4.39	23.63	56.47	10	32.26	2.61	17.32	51.47	12	85.71	14.36	56.15	97.48
Toxascaris leonina	1	2.78	0.03	0.15	16.21	1	3.23	0.03	0.17	18.52	1	7.14	0.07	0.37	35.83
Ancylostoma tubaeformae	6	16.67	0.92	6.97	33.47	7	22.58	2.45	10.28	41.54	4	28.57	0.93	9.58	58.00
Taenia taeniaeformis	13	36.11	1.78	21.34	53.79	13	41.94	2.45	25.07	60.74	8	57.14	4.86	29.65	81.18
Dipylidium caninum	1	2.78	0.03	0.15	16.21	2	6.45	0.16	1.12	22.84	2	14.29	0.57	2.52	43.85
Mesocestoides sp.	0	0.00	0.00	0.00	12.01	2	6.45	0.06	1.12	22.84	0	0.00	0.00	0.00	26.76
Eucoleus aerophilus	0	0.00	0.00	0.00	12.01	2	6.45	0.16	1.12	22.84	0	0.00	0.00	0.00	26.76
